# Nanostructured Thin Films Enhancing the Performance of New Organic Electronic Devices: Does It Make Sense?

**DOI:** 10.1021/acsmaterialsau.4c00103

**Published:** 2024-10-31

**Authors:** Priscila Alessio, Milene K. C. da Silva, Vitoria Barossi, Celina M. Miyazaki

**Affiliations:** † Department of Physics, School of Technology and Sciences, São Paulo State University (UNESP), Presidente Prudente, SP 19060-080, Brazil

**Keywords:** nanostructured thin films, organic electronics, sustainable sensors, flexible sensors, biodegradable sensors, paper-based sensors

## Abstract

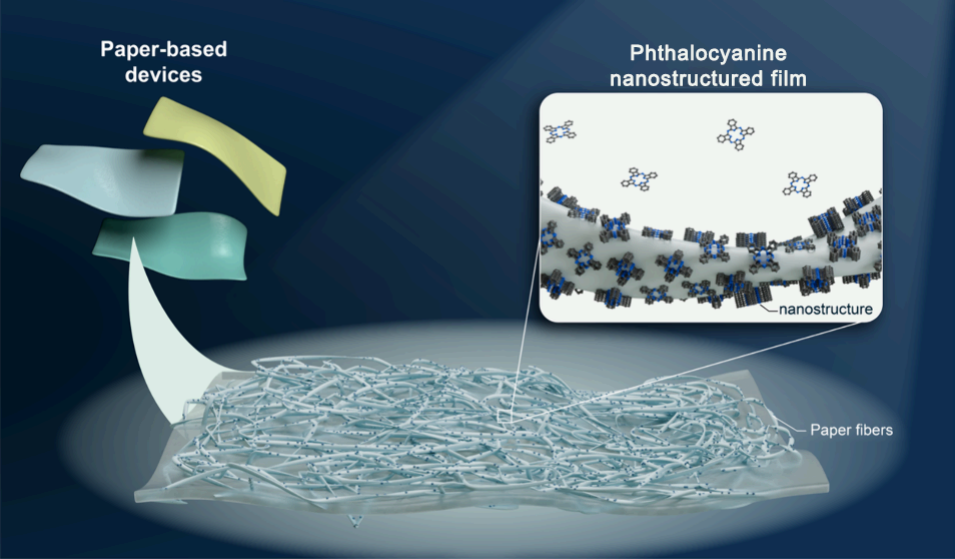

Electronics have evolved significantly with the development of semiconductor materials and devices, with emerging areas such as organic and flexible electronics showing great promise, particularly in applications such as wearable devices and environmental sensors. Since the discovery of conducting polymers in the late 1970s, organic electronics have paved the way for innovations such as organic field-effect transistors (OFETs), organic light-emitting diodes (OLEDs), and organic solar cells (OPVs). Recent advances have focused on nanostructuring techniques to enhance device properties, such as charge mobility and luminescence efficiency. The growing concern for sustainability has also led to the exploration of biodegradable organic electronics as a potential solution to electronic waste. This perspective briefly discusses the impact of nanostructuring on the performance of both conventional and biodegradable organic devices, exploring the challenges and opportunities associated with using alternative substrates like paper. This perspective emphasizes the importance of understanding molecular organization at the nanoscale to optimize device performance and ensure stability under practical conditions.

## 1 Introduction

Modern electronics encompasses the development and advanced applications of semiconductor materials, devices, and circuits aimed at acquiring, processing, controlling, and transmitting information efficiently. Some subareas, such as organic electronics and flexible electronics, stand out as emerging, especially in wearable devices, flexible displays, and medical and environmental sensors. These subareas have the potential to revolutionize several industries mainly due to the ability to develop flexible, lightweight, and low-cost devices, which provides these subareas with numerous advantages over traditional electronics.

**Figure 1:**
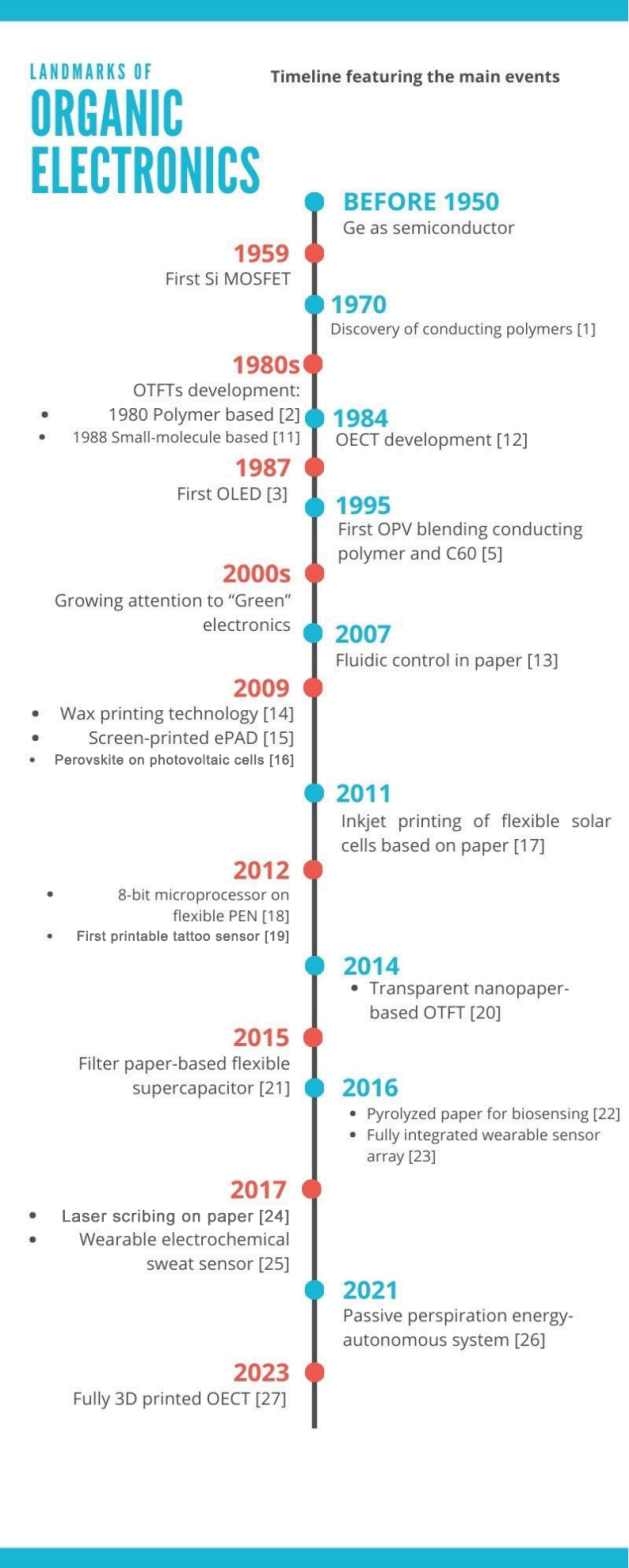
Timeline featuring the main events of organic electronics.^10−26^

Since the discovery of conducting polymers in the late 1970s by Heeger, MacDiarmid, and Shirakawa,^1^ who received the Nobel Prize in Chemistry in 2000 for this work, the path for the development of organic electronics has been paved. This discovery paved the way for the development of several organic electronic devices, beginning in the 1980s with research that resulted in the creation of organic field-effect transistors (OFETs).^2^ The 1990s saw the introduction of organic light-emitting diodes (OLEDs),^3^ which quickly found applications in displays and lighting. After the first publication in 1995,^4^ organic solar cells (OPVs) gained prominence in the 2000s due to their potential for low-cost renewable energy. In parallel, organic sensors have emerged, taking advantage of the flexibility and functionalization capacity of organic materials for applications in environmental monitoring and biomedical diagnostics. More recently, the integration of organic electronics with flexible and printable electronics has driven the creation of wearable devices, advanced medical sensors, and other innovative applications, marking a new era of sustainable and versatile technological possibilities.^5,6^Figure 1 illustrates a summary of the historical evolution reports related to the organic electronic development, mentioned above.

This rapid evolution of technologies is driven by significant advances in the areas of organic material synthesis and manufacturing techniques. These advances provided more precise control over the materials properties, which play an important role in optimizing the device’s performance control. Nanostructuring or nanoarchitectonics, in particular, has proven to be a promising approach to improving the properties of organic electronic devices. This is because the possibility of controlling the morphology and organization of materials at the nanometric scale allows for optimization of characteristics such as charge mobility, luminescence efficiency, and device stability. This possibility to fine-tune materials characteristics using a bottom-up approach makes nanoarchitectonics an essential step for the development of functional materials to application in devices to a wide range of applications, such as sensors, light-emitting devices, bioimaging, energy storage, and neuromorphics, among others.^7−9^

On the other hand, the evolution of electronics is currently facing growing concerns about environmental awareness, sustainability, and the need to reinvent itself. Now, the development of devices needs to consider not only the final efficiency but also environmentally friendly production and disposal technologies. Thus, biodegradable organic electronics have emerged as a potential solution, with the ability to significantly reduce the environmental impact of electronic waste.

This perspective aims to briefly explore the possibilities of biodegradable organic electronics, discussing the possible importance and influence of nanostructuring on the properties of biodegradable devices and the associated challenges. In addition, the types of substrates used and the characterization techniques of nanostructures are concisely discussed, offering a critical view of the current state and future directions of organic electronics research.

## 2 Nanostructured Film Role for Conventional Organic Electronics

Nanostructuring plays a crucial role in optimizing the properties of organic electronic devices, which we will call as conventional here, i.e., those built on flat rigid glass and silicon substrates. By modifying the surface with thin films with controlled molecular organization and morphology at the nanometric scale, it is possible to significantly improve the performance of these devices, allowing them to achieve optimized operation conditions. This improvement in the performance can be attributed to several key mechanisms. For instance, by controlling the morphology and molecular organization of materials at the nanoscale, nanostructuring optimizes charge transport pathways, reduces recombination losses, and increases the surface area for active material interactions. These adjustments lead to an enhanced charge mobility, higher luminescence efficiency, and greater device stability. Studies have shown that such nanoscale organization enables more efficient energy transfer and charge separation, which collectively result in the improved operation of organic electronic devices.^7,8^

In the literature, it is possible to find works addressing the role of nanostructuring in several properties, such as charge mobility, conductivity, conversion efficiency, emission efficiency, and stability and durability.^27−29^ Some examples are OFETs with nanostructured channels that exhibit higher charge mobilities, basically due to nanostructuring, which create more efficient paths for electron and hole mobility, reducing dispersion and increasing device efficiency.^30^

Other work shows that OPVs with bulk heterojunction structures present improvements in power conversion efficiency due to nanostructuring of the active layer, which facilitates charge separation and efficient collection of electrons and holes.^31^ Nanostructuring of emitting materials in OLEDs can increase quantum efficiency, improving color intensity and purity.^32^ The formation of excitonic states (electron–hole pairs) that recombine radiatively can be optimized by controlling the nanoscale morphology of the emitting materials. Another point that nanostructuring can contribute is the reduction of emission “quenching”, which occurs due to interactions between emitting molecules. Nanometric structures can space these molecules, reducing nonradiative recombination.^33^

Self-assembled nanocrystalline thin films have also been the focus of diverse organic electronic devices.^34−39^ Nanostructures and nanolayers with hierarchical growth can be obtained by controlling experimental parameters (e.g., proportions of different surfactants and solvents),^40,41^ enabling control between π–π stacking and steric hindrance of functional groups, which can be intentionally inserted into the organic chain skeleton. However, usually these organized nanostructures are organic solvent assisted.^38,41^ Strategies have been thought up for greener synthesis, utilizing water and water–alcohol processing.^37,42,43^ These eco-friendly solvent alternatives can drive this technology to the transition to biodegradable substrates, susceptible to degradation under aggressive solvent conditions.

Nanostructuring is especially important for materials with anisotropic properties, as is the case for two well-known carbon structures in organic electronics: graphene and carbon nanotubes. The electronic conductivity of graphene is significantly higher in-plane than cross-plane, while the conductivity of nanotubes is higher along their axis. Although techniques such as CVD provide organized thin films, their transfer to alternative substrates can be challenging. When transferred to rigid flat and cylindrical glasses, a CVD-grown CNT-based device exhibited similar *I–V* curves, indicating that the film can conform. In the transfer for PET and PEN, the substrates suffered little damage by acetone solvent, and the resistance of the device was 2–3 times that of the glass-based device. Paper-based devices presented a much-reduced current, which was explained by the loss of intimate contact of the CNTs and substrate due to the high roughness and the different electrostatic environment.^44^ Although exemplified here by CNTs, the concern with achieving the transfer of organized structures (exactly as those obtained in glass) to porous and heterogeneous surfaces extends to other materials of interest in organic electronics.

Some research groups are dedicated to conducting studies of the relationship between nanostructuring and properties. A series of studies discuss the molecular arrangement of molecules as a function of the nanofabrication technique,^45−48^ the influence on photoluminescence,^47,48^ and electrical conductivity.^46^ Nanostructuring techniques such as PVD, Langmuir–Schaefer (LS), and Langmuir–Blodgett (LB) techniques were explored by the authors using homogeneous substrates, such as glass and quartz.

**Figure 2:**
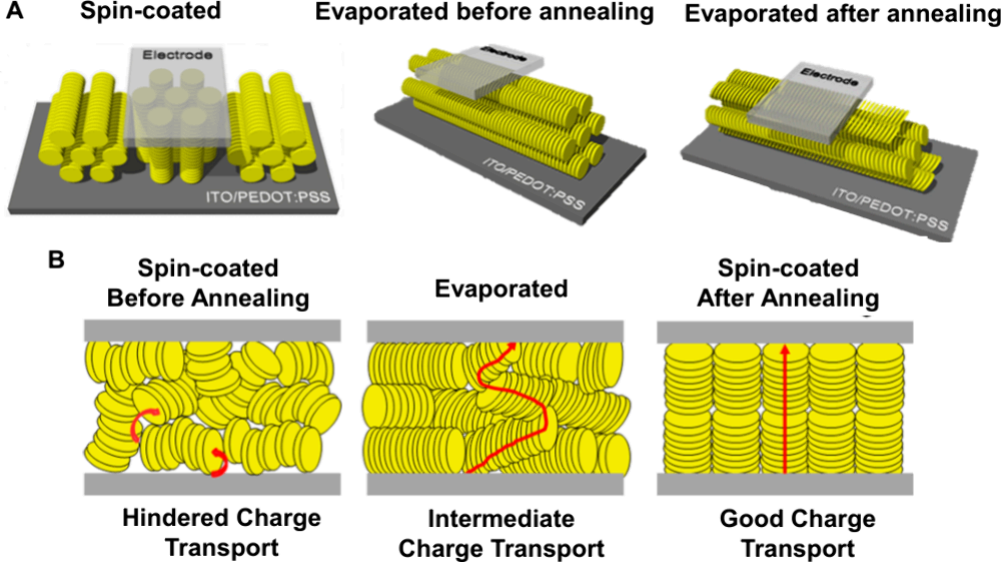
Schematics of (A) molecular organization of the spin-coated CoILC film in the edge-on orientation (open film) and face-on orientation (under the electrode) and evaporated CoILC film before and after annealing. (B) Charge transport of different molecular packing: (a) spin-coated before annealing, (b) evaporated, and (c) spin-coated after annealing. Reprinted with permission from (49). Copyright 2015 American Chemical Society.

Charge transport is an important property for several devices, and in thin films, it varies depending on the nanofabrication technique.^49^ For instance, spin-coated films of columnar liquid crystals (ColLCs) based on the perylene diimide core have charge transport hampered by the low packing. After annealing, the molecules acquire face-on orientation (homeotropic alignment) under the area of the metallic contact, with improved charge transport (Figure 2A). On the other hand, evaporated films have dense packing that does not allow for reorientation during heat treatment, and the columnar films remain edge-on oriented, with intermediate charge transport between the pre- and post-annealing spin-coated films (Figure 2B). Given such results regarding the orientation–performance relationship, it is curious what to expect from molecular conformation/orientation on complex heterogeneous and porous surfaces.

Systematic studies of molecular conformation and organization on the substrate help us to understand the desired (or undesired) performance of the devices. These investigations usually combine in-depth morphological and/or spectroscopic analyses of thin films of supramolecules, mainly phthalocyanines and perylenes, deposited onto conventional substrates, but to date, organization on unconventional substrates (with high porosity and fibrous structures) has not yet been deeply investigated. In the specific case of paper, what should we expect from a highly porous and heterogeneous material? Could some organized arrangement within the fibers be expected? Should we expect a “conventional” nanostructuration above a certain thickness? Up to the present time, the literature has addressed deposition methods via drop casting. However, detailed studies dealing with more sophisticated deposition methods (allowing organizational control) will help to answer these questions and predict and improve the performance of these new promising flexible and biodegradable electronic devices.

## 3 Sustainable and Biodegradable Electronics

**Figure 3:**
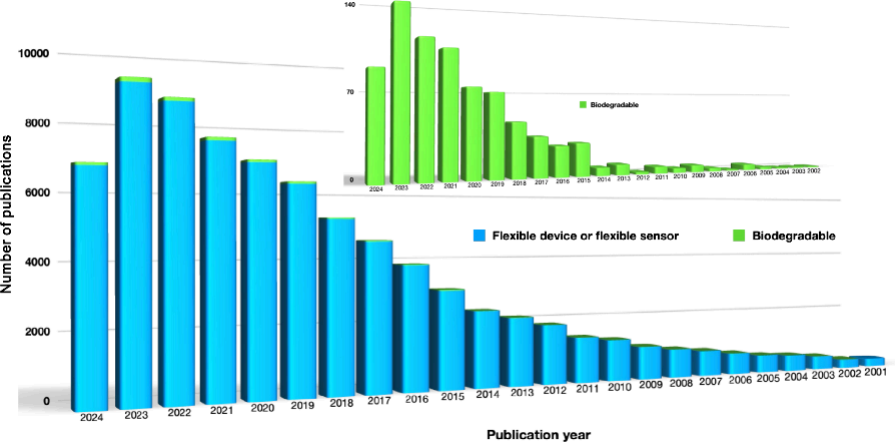
Number of publications on “flexible device or flexible sensor” is shown in blue and refined with “biodegradable” in green over time. The inset shows the biodegradable data on an enlarged scale. Data obtained from Web of Science, accessed on August 27, 2024 and available at https://www.webofscience.com/wos/woscc/summary/06030ef0-98bd-44f3-9579-0455d7bb883a-01045e6b95/relevance/1.

Although flexible devices and sensors have shown great promise in organic electronics, only a small portion of studies have explored biodegradable devices. Figure 3 summarizes the result of a search on Web of Science and shows that of the more than 79 000 publications addressing flexible devices and flexible sensors, only ∼770 publications address biodegradable devices and sensors. It is evident that the research area of biodegradable devices is growing (Figure 3, inset), and it presents a promising opportunity for researchers to make significant contributions to sustainable technology and pave the way for innovative solutions that align with global sustainability goals.

To replace conventional glass-based devices with flexible devices, polymeric substrates such as PET (polyethylene terephthalate) and PEN (polyethylene naphthalate) are good candidates due to their controllable transparency, mechanical stability, chemical resistance, and low permeability to gases and water vapor. These polymers are processable on a large scale and have favorable mechanical and optical properties. Comparatively, paper substrates are attractive for flexible electronics due to their biodegradability and low cost, but their rough surface and limited thermal and chemical resistance restrict their use. In terms of performance, polymers outperform paper in stability and compatibility with solution processes, while paper offers ecological and economic advantages.

Nanocellulosics are emerging as efficient alternatives to plastic substrates in electronic sensors, offering billions of sensory elements at low environmental and economic costs. There is growing interest in the development of sensors and sensory systems using nanocellulosic materials, such as nanocellulose-enhanced organohydrogels, which exhibit high strength, conductivity, transparency, and antifreeze properties for wearable sensors. These materials, which replace fossil-based conductive polymers, demonstrate self-healing and material recovery capabilities after use. In addition, OLEDs and other flexible optoelectronic devices made with nanocellulose offer advantages in terms of flexibility, thermal stability, and optoelectric efficiency compared to conventional plastics, standing out as a promising trend for sustainable development.^50^

While natural materials still exhibit some drawbacks related to high processing costs and high batch-to-batch variation, biodegradable and biocompatible synthetic materials have proven to be good alternatives for flexible, wearable, and eco-friendly devices, such as polyurethane (PU) and poly(lactic acid) (PLA). Biodegradability is also a characteristic of these polymers, which increases the importance of related studies, since the disposal of electronic waste (e-waste) is becoming increasingly worrying. PLA is a thermoplastic polymer from renewable sources, approved by the FDA (Food and Drug Administration), and widely used in the packaging and biomedical industries. As an alternative substrate, it must accommodate electrodes that are mostly placed via spin-coating and printing techniques. Ensuring a degree of organization on these substrates can be a challenge because of the already mentioned points, i.e., experimental conditions can end up degrading the polymer (high temperature, pressure, and solvents). In this sense, milder methods such as Langmuir–Blodgett and Langmuir–Schaefer methods can become great allies, although studies are needed to understand how organized monolayers on the air–water interface are transferred to porous and heterogeneous substrates.

Paper-based analytical devices (PADs) or μPADs, due to their inherent porous characteristic that allows liquids to move by capillarity, have attracted great attention in the areas of energy^51^ and sensors.^52,53^ In a very simplified way, and taking ePADs (electrochemical paper-based analytical devices) as an example, the challenges are centered on the definition of the hydrophobic regions that delimit the “wet” area and the placement of the electrodes on the paper to achieve sufficient conductivity, mechanical stability, and reproducibility in electrochemical measurements in the face of natural variations in the paper and the presence of processing residues that become contaminants in the analyses.

While paper capillarity is extremely necessary to promote the movement of reagents and analytes in μPADs, it is a problem in the manufacture of printed circuit boards (PCBs). The liquid reagents penetrate the pores of the paper and can disperse to undesired regions, leading to difficulty of producing tracks with high definition and generating short circuits between conductive tracks. Working out issues related to low stability in contact with water and high roughness is in demand for implementing general papers in electronics. Applying sequential layers of waterproof encapsulation and flexible planarization coating materials could achieve this. The encapsulation is made with wax and protects against the penetration of oxygen and liquids. The planarization layer of polysilsesquioxane reduces surface roughness.^54^ An interlayer of sublimated copper phthalocyanine (CuPc) organic thin films between the aluminum tracks and paper enables the production of a PCB, improving the adhesion of aluminum particles and reducing the porosity of the paper.^55^

In addition to the advantages of the paper substrate already mentioned, such as sustainability, flexibility, and recyclability, paper has also been studied for its potential to act as the active material of the device.^56^ Techniques such as pyrolysis (thermal decomposition in the absence of oxygen) and LIG (laser-induced graphene) allow for the generation of conductive electrodes and tracks directly on paper (and other carbon-rich substrates). Although promising, major challenges exist that need to be addressed before these electrodes achieve reproducibility and the necessary conductivity. Another challenge focuses on the modification of these electrodes. Although it is easily performed on conventional substrates, the deposition of thin films via wet processes can be challenging in these devices, considering drawbacks such as electrode peeling and irregular film deposition due to the swelling effect and variation in the wettability of the heterogeneous surface. Knowing that the functionalization and use of nanostructured materials can significantly improve charge transport and sensor sensitivity, it is of utmost importance to understand how nanostructuring occurs in these new materials to establish adequate experimental protocols like those well-established for the functionalization of conventional electrodes.

In the matters of biodegradability, a device’s lifespan should align with its function, such as matching tissue regeneration timelines in medical applications to avoid invasive removal.^57^ The degradation products must be nontoxic and naturally eliminated. Understanding the degradation mechanisms is essential to define the device’s lifespan. Passively transient devices lose functionality as they degrade, while others can be triggered by controlled conditions when no longer needed.^58^ Biodegradable materials, often polymers, should be used as substrates due to their significant impact on the device’s overall degradation. Natural polymers like cellulose and chitosan degrade enzymatically but face challenges such as batch-to-batch variation and bioactivity, potentially causing immunogenic responses.^59^ Degradation occurs through oxidation and hydrolysis. Synthetic polymers are vulnerable to hydrolysis through ester, imides, amides, ethers, imines, thioesters, sulfonamides, anhydrides, phosphonates, carbonates, urethanes, and ureas.^57^ The degradation rate depends on factors such as polymer morphology and crystallinity. A 2021 study introduced a biodegradable electrochemical glucose biosensor using silkworm fibroin with platinum electrodes and biocompatible silicone. The sensor fully degraded by day 10 under enzymatic action.^60^ Cellulose-based substrates are widely explored due to their abundance and flexibility, with various degradation methods, including UV and enzymatic catalysis.^61^

## 4 Case Study: Phthalocyanine Thin Film Nanostructuring on Glass versus Paper

Several techniques can be used to investigate the molecular organization in thin films. For instance, grazing incidence wide-angle X-ray scattering (GIWAXS) is a gold standard technique to study the crystalline structure and orientation of molecules within thin films.^49^ Other widely used techniques are polarized Raman spectroscopy, which can provide information on molecular orientation by analyzing the polarization dependence of the Raman signal,^62^ and polarized infrared reflection absorption spectroscopy (IRRAS), which can reveal the orientation of molecular bonds in thin films. Surface-enhanced Raman spectroscopy (SERS) enhances Raman signals near metallic nanostructures, allowing for detailed analysis of molecular orientation and organization in thin films.^63^ It is noteworthy that each technique requires an adequate substrate.

**Figure 4:**
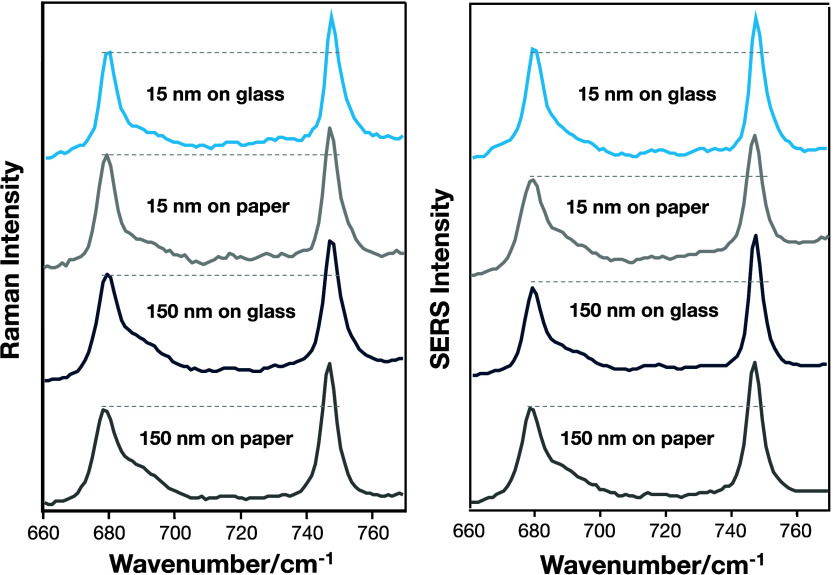
Relative intensity of C–H_in-plane_ and C–H_out-of-plane_ vibration modes obtained for the RRS and SERRS spectra for the CuPc PVD film with 15 and 150 nm thicknesses on glass and paper. The SERRS measurements were carried out using silver nanoparticles dropped and dried on the film surface.

Thus, to investigate the potential nanostructuring of organized films on paper substrates, we conducted a case study with films of CuPc obtained by using PVD by thermal evaporation. The CuPc PVD films (150 nm) were deposited on different substrates: ZnSe, Au mirror, glass, and filter paper. The molecular organization of the film was investigated by FTIR allied to the selection rules, as described in detail in refs (64) and (65) for the films deposited on the ZnSe and Au mirror. Complementary resonant Raman spectroscopy and SERRS (surface-enhanced resonant Raman spectroscopy) were utilized to investigate the molecular organization on the glass and paper substrates through the ratio between the C–H in-plane and C–H out-of-plane vibration intensities, as shown in Figure 4.

The ratio *I*_679_/*I*_745_ (C–H in-plane/C–H out-of-plane) indicates a tendency of the CuPc molecules to organize themselves with the macrocycle perpendicular to the substrate surface, which is in full agreement with the FTIR measurements (not shown). Thus, the FTIR and Raman results point out that the same molecular organization is on average obtained for the different substrates. The CuPc molecules organized themselves preferentially in a perpendicular arrangement, which agrees with the literature, where an angle of ∼65° with respect to the substrate was reported for thermally evaporated films.^66^ Yet, the film thickness (15 or 150 nm) did not influence the organization of the studied system. However, further investigation on the nanoscale needs to be conducted to verify the influence of the substrate’s porosity, fiber structures, stretchability, surface roughness, chemical composition, and film thickness on the nanostructuration. On the other hand, the performance of the devices should also be evaluated.

## 5 Final Remarks

In this work, we highlighted the significant role of nanometer-scale organization in determining the electronic and optical properties of conventional devices. We emphasize the growing importance of studying molecular organization in emerging materials for electronics, particularly those involving flexible and biodegradable substrates. Understanding this nanostructuration is essential not only for optimizing device performance but also for ensuring the stability of these materials under practical conditions such as repeated mechanical deformations in flexible substrates or exposure to moisture in highly porous and fibrous materials like paper. Continued research in these areas is crucial for advancing the development of sustainable, high-performance electronic devices.

## Abbreviations

CNTs: carbon nanotubes
ColLCs: columnar liquid crystals
CVD: chemical vapor deposition
CuPc: copper phthalocyanine
ePAD: electrochemical paper-based analytical devices
GIWAXS: grazing incidence wide-angle X-ray scattering
IRRAS: infrared reflection absorption spectroscopy
LIG: laser-induced graphene
LB: Langmuir–Blodgett
LS: Langmuir–Schaefer
MOSFET: metal-oxide semiconductor field-effect transistor
OECT: organic electrochemical transistor
OFETs: organic field-effect transistors
OLEDs: organic light-emitting diodes
OPVs: organic photovoltaics
OTFT: organic thin-film transistor
PADs: paper-based analytical devices
PCBs: printed circuit boards
PEN: polyethylene naphthalate
PET: polyethylene terephthalate
PLA: poly(lactic acid)
PVD: physical vapor deposition
RRS: resonant Raman scattering
SERS: surface-enhanced Raman spectroscopy
SERRS: surface-enhanced resonant Raman spectroscopy
